# Severe thyrotoxicosis as initial presentation of gastric choriocarcinoma: a case report

**DOI:** 10.1186/s13256-022-03343-5

**Published:** 2022-04-21

**Authors:** Nicole M. Iñiguez-Ariza, Dalia Cuenca, Juvenal Franco-Granillo, Alberto Villalobos-Prieto, Janet Pineda-Díaz, Javier Baquera-Heredia

**Affiliations:** 1 Department of Medicine, American British Cowdray Medical Center, Mexico City, Mexico; 2 Department of Endocrinology and Metabolism, Instituto Nacional de Ciencias Médicas y Nutrición Salvador Zubirán, Mexico City, Mexico; 3 Critical Care Unit, American British Cowdray Medical Center, Mexico City, Mexico; 4 Department of Medical Oncology, American British Cowdray Medical Center, Mexico City, Mexico; 5 Department of Surgical and Molecular Pathology, American British Cowdray Medical Center, Mexico City, Mexico

**Keywords:** Hyperthyroidism, Paraneoplastic syndrome, Human chorionic gonadotropin, Germ cell tumor, Case report

## Abstract

**Background:**

Extragonadal choriocarcinoma is rare and can be associated with hyperthyroidism when producing very high levels of human chorionic gonadotropin.

**Case presentation:**

A 62-year-old Hispanic female presented with a 3-week history of shortness of breath, palpitations, extreme weakness, new-onset hot flashes, and right flank pain. Her physical examination was remarkable for tachycardia, hepatomegaly, hyperreflexia, and tremor; goiter was absent. Laboratory studies revealed increased lactate dehydrogenase, alkaline phosphatase, suppressed thyroid stimulating hormone, very elevated T4, and absent thyroid stimulating immunoglobulin. ^18^F-fluorodeoxyglucose positron emission tomography-computed tomography exhibited hepatomegaly with multiple large fluorodeoxyglucose-avid liver masses and a focus of fluorodeoxyglucose avidity in the stomach with no structural correlate. A thyroid scan (^99m^TcO_4_^−^) showed diffusely increased tracer uptake. She was started on propranolol and methimazole. Upon stabilization of severe thyrotoxicosis, upper endoscopy was performed, showing a ~ 5 cm bleeding lesion in the greater stomach curvature body; biopsy was consistent with choriocarcinoma; beta-human chorionic gonadotropin hormone was 2,408,171 mIU/mL. The patient received methotrexate followed by etoposide and cisplatin. Methimazole was titrated down, and upon liver failure the medication was stopped. The thyrotoxicosis was effectively controlled with antithyroid drug and concurrent chemotherapy. At ~ 1.5 months after initial diagnosis, the patient died due to bleeding/acute liver failure with coagulation defects followed by multiple organ failure.

**Conclusions:**

Severe thyrotoxicosis can represent an unusual initial presentation of metastatic choriocarcinoma in the setting of extreme elevation of beta-human chorionic gonadotropin. Primary gastric choriocarcinoma is an aggressive malignancy with very poor outcomes. The co-occurrence of severe thyrotoxicosis with advanced primary gastric choriocarcinoma and imminent liver failure complicates management options.

## Introduction

Choriocarcinoma is a human chorionic gonadotropin (hCG)-producing tumor observed in the setting of gestational trophoblastic disease (GTD) [1]. GTD entails a group of benign (hydatiform mole) and malignant (invasive mole, choriocarcinoma, and other) tumors arising from the uterus/placenta. Choriocarcinoma represents < 5% of all GTDs and can be associated with very high hCG concentration capable of inducing hyperthyroidism due to cross-reactivity with the TSH receptor. GTD patients usually enjoy excellent prognosis in response to effective chemotherapy (long-term survival > 95%), but high-risk malignant GTD lessens 5-year overall survival (OS, 75–90%) [2, 3].

Choriocarcinomas rarely occur in extragonadal sites (e.g. lung, gastrointestinal tract, mediastinum, retroperitoneum, and other) with primary gastric choriocarcinoma (PGC) representing 0.08% of all gastric cancers [4, 5]. However, unlike gonadal choriocarcinoma, PGC is associated with very poor prognosis with median OS of < 2 months [5–7]. Herein, we present a case of a patient with metastatic PGC presenting with thyrotoxicosis.

## Case presentation

A previously healthy 62-year-old Hispanic gravida 5 woman (2 spontaneous abortions), presented with a 3-week history of increasing shortness of breath, palpitations, extreme weakness, new-onset hot flashes, and right flank pain. Physical examination was remarkable for tachycardia (heart rate ~ 100 ×), temperature of 37.7 °C, speech compromised by shortness of breath, eyes with pale conjunctivae (with no erythema, chemosis, or exophthalmos), thyroid gland normal to palpation without nodules (~ 15 g), heart sounds increased in frequency with a systolic tricuspid murmur, basal lung breath sounds decreased to auscultation, hepatomegaly of ~ 5 cm below the right costal margin extending to the epigastrium (with hardness and tenderness to palpation in this area), as well as hyperreflexia and tremor. Chest X-ray and renal ultrasound demonstrated normal lung and renal findings but multiple liver nodules.

**Fig. 1:**
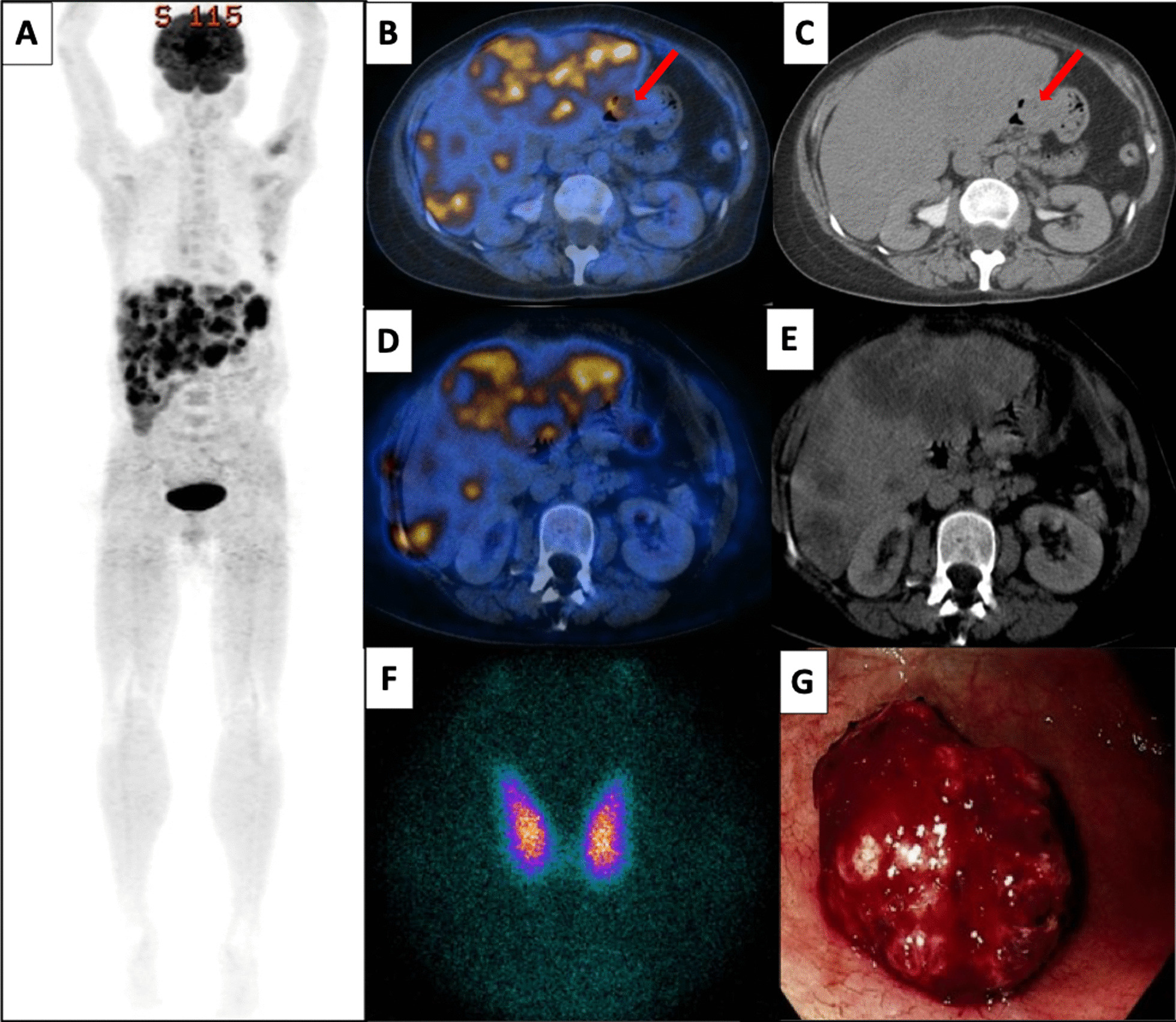
Imaging studies upon presentation and upper endoscopy. **A**–**E** Contrast-enhanced ^18^FDG PET-CT exhibited hepatomegaly (22 cm longitudinal axis) with multiple large FDG-avid liver masses, measuring up to 12 cm and SUV max of 13.5. A focus of FDG avidity in the gastric antrum had no structural correlate (red arrows), with an SUV max of 9.4. CT also showed mesenteric lymph nodes and small lung infiltrates. **F** Thyroid scan (^99m^TcO_4_^−^) with tendency to diffusely increased tracer uptake (radioiodine thyroid scan was not performed due to use of IV-iodinated contrast in the preceding two days). **G** Upper endoscopy showing a ~ 5 cm bleeding lesion in the greater stomach curvature body

She was referred to our institution for evaluation and management. Laboratory tests indicated normocytic anemia (hemoglobin 8.1 g/dL), elevated lactate dehydrogenase at 3054 U/L (122–222), alkaline phosphatase 434 U/L (40–29), and elevated T4 with suppressed TSH consistent with thyrotoxicosis (Table 1). Her echocardiogram showed pulmonary hypertension (65 mmHg), dilated right atrium and ventricle, tricuspid regurgitation, adequate left ejection fraction, and a previously unknown atrial septal defect with inter-auricular communication with a right to left shunt and high risk for embolism. Contrast-enhanced ^18^FDG PET-CT showed hepatomegaly with multiple large FDG-avid liver masses, with a focus of FDG avidity in the stomach with no structural correlate (Fig. 1).

**Table 1:** Laboratory tests and treatment modalities

	Upon presentation	At 10 days post MTX dose and 2 weeks post ATD initiation	At 4 weeks post ATD initiation	At readmission^a^	At 10 days post EP^b^
FT4 (0.71–1.85 ng/dL)	4.1	1.5	1.1	1.1	1.1
TT4 (4.5–12 mcg/dL)	30.2	9.5	7.4	5.2	–
TSH (0.45–5 mIU/L)	0.06	0.04	0.01	0.02	0.07
β-hCG* (0–10 mIU/mL)	2,408,171	3,904,164	–	3,105,708	832,558
Methimazole dose (mg/day)	30	20	10	Stopped	–

*TSH* thyroid stimulating hormone, *FT4* free thyroxine, *TT4* total thyroxine, β-hCG* beta-human chorionic gonadotropin hormone, confirmed by serial dilution up to 1:100, *MTX* methotrexate, *ATD* antithyroid drug, *EP* etoposide-cisplatin

Concurrent with first chemotherapy (MTX) she received not only ATD but also Lugol’s solution for a couple of days due to threat of transient increase in β-HCG levels and consequent worsening thyrotoxicosis

^a^During her readmission, she was treated aggressively in the intensive care setting, her AST and ALT trended down from 1346 U/L and 500 U/L, respectively, to 247 U/L and 110 U/L, respectively

^b^At 11 days post-EP, the patient died due to multi-organic failure, particularly liver failure (AST 7224 U/L, ALT 2026 U/L, total bilirubin 27.4 mg/dL) with disseminated intravascular coagulation and persistent gastrointestinal bleeding

Her thyroid function tests and thyroid scan with technetium 99m pertechnetate (^99m^TcO_4_^−^) were consistent with thyrotoxicosis due to hyperthyroidism (Table 1; Fig. 1). Graves’ disease was ruled out in the setting of no signs of Graves orbitopathy, no goiter, and negative thyroid stimulating antibodies (TSI index < 1.0 and thyroid receptor antibody < 1.00 IU/L). At presentation, she also had mildly altered liver function tests [AST 137 U/L (10–50), ALT 70 U/L (8–54), total bilirubin 1.8 mg/dL (0.3–1.3].

**Fig. 2:**
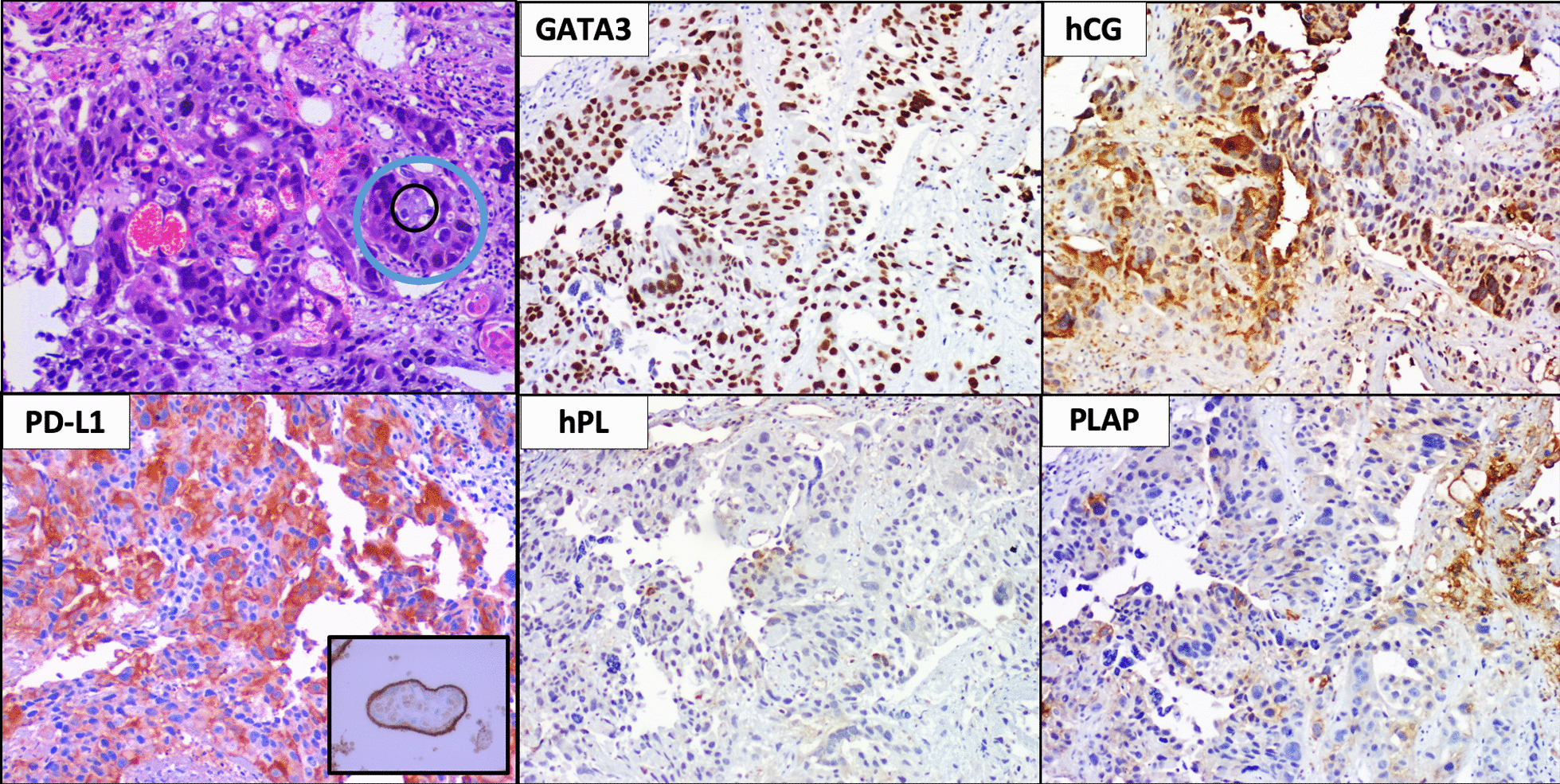
Histopathology biopsy results. Upper-left image: The microscopic field shows the morphologic features of the neoplasm with a biphasic growth pattern with pleomorphic multinucleated cells (blue circle) surrounding islands of basophilic, mononucleated cells (black circle) in a lacunar vascular pattern. (Hematoxylin & eosin, 100×). The other images show the tumor immunoprofile: GATA3+, hCG+, hPL, and PLAP focally positive, supporting the diagnosis of choriocarcinoma. Neoplastic cells had strong and diffuse membranous reactivity to PD-L1 antibody, as normal placental villous tissue does (inset)

She was started on propranolol and antithyroid drug (methimazole 30 mg/day). Upon stabilization of severe thyrotoxicosis, upper endoscopy was performed, showing a ~ 5 cm bleeding lesion in the greater stomach curvature (Fig. 2). Gastric biopsy was consistent with choriocarcinoma with extensive necrosis with no evidence of concurrent conventional adenocarcinoma nor endodermal sinus component; immunostaining with antibodies against hCG, GATA3, LPH, PLAP, and CK7 was positive (Fig. 2). Neoplastic cells had strong and diffuse membranous reactivity to PD-L1 antibody.

Subsequently, beta-human chorionic gonadotropin hormone (β-hCG) was found to be extremely elevated at 2,408,171 mIU/mL. Other tumor markers (CA-125, CEA, and AFP) were measured and unrevealing. After this workup, an extragonadal germinal tumor (primary gastric choriocarcinoma) with extensive liver metastases and paraneoplastic hyperthyroidism was deemed the most likely diagnosis.

The patient was treated initially with methotrexate (200 mg/m^2^) and subsequently with etoposide and cisplatin. Methimazole was titrated down from 30 to 10 mg/day, achieving euthyroidism. The thyrotoxicosis was effectively controlled with antithyroid drug and concurrent chemotherapy.

One month after initial diagnosis, the patient was readmitted with hemorrhagic hypovolemic shock due to gastrointestinal bleeding and acute liver failure with severe coagulopathy (AST 1346 U/L, ALT 500 U/L, total bilirubin 5.6 mg/dL, altered coagulation: INR 2.17, fibrinogen 111 mg/dL). Methimazole was discontinued upon liver failure onset. She required intensive care therapies with a multidisciplinary team, but unfortunately died ~ 1.5 months post presentation in the setting of multiple organ failure.

## Discussion

Primary gastric choriocarcinoma (PGC) occurs more frequently in males (2.3:1) at a median of 63 years of age. The mean tumor size at diagnosis is 7 cm. The most common location is in the lower third of the stomach (40%), with almost all having macroscopic hemorrhage or necrosis [4]. Our case is consistent with this clinical presentation, with the exception of gender and gastric location. PGC is a very rare and extremely aggressive tumor that, unlike primary gastric adenocarcinoma (which usually metastasizes through lymphatics), is characterized by rapid proliferation and early distant hematogenous spread [5, 8]. In a pooled analysis of 53 PGC cases, most presented with metastatic disease, with liver metastases occurring in 45% [4]. In the present case, the primary tumor size was ~ 5 cm, while the liver had disseminated metastatic disease with some of the liver metastases as large as ~ 10 cm.

The etiology of PGC is debated. The less accepted theory is that PGC occurs de novo, since hCG-producing cells have been found in normal gastric mucosa and gastric adenocarcinoma; the existence of these cells might be the origin of pure forms of PGC, without preceding adenocarcinoma. Nonetheless, the most accepted theory is the “dedifferentiation theory,” with dedifferentiation of gastric adenocarcinoma to the embryonal ectoderm level, but with the ability to form trophoblasts. Supporting the latter theory is the fact that most PGC have a poorly differentiated adenocarcinoma component (~ 70%); moreover, PGC genetically has characteristics of both adenocarcinoma and gestational choriocarcinoma [4, 5, 9]. The dedifferentiation theory suggests that pure PGC occurs when overgrowth and elimination of the original adenocarcinoma has occurred [5, 10].

Our case is a histologically pure PGC, although we cannot with certainty exclude a possible adenocarcinoma component in unsampled tumor areas. Although definitive diagnosis by endoscopic biopsy is rare (8%), we were able to achieve a biopsy-proven diagnosis [5].

The differential appropriate diagnosis of the etiology of recent-onset thyrotoxicosis is of particular importance in the sense that it significantly impacts treatment choice strategies. Thyrotoxicosis can occur in the context of increased thyroid function, release of preformed hormones, or even because of factitious ingestion of thyroid hormones. History and physical examination (e.g., orbitopathy, goiter), measurement of thyroid stimulating antibodies, and nuclear medicine thyroid scans (radioiodine or ^99m^TC pertechnetate) are of aid in the initial approach to thyrotoxicosis [11].

Though β-hCG-mediated thyrotoxicosis is a well-known but infrequent cause of overt hyperthyroidism, the typical clinical scenario is early pregnancy or recent obstetric events (pregnancy, abortion). The diagnosis of β-hCG-mediated hyperthyroidism in a ~ 60-year-old woman is quite a challenge, and the finding of PGC with paraneoplastic hyperthyroidism as the cause of her thyrotoxicosis was surprising.

Biochemical hyperthyroidism during pregnancy occurs most commonly due to gestational hyperthyroidism (GH) or Graves’ disease. The former usually appears in the first trimester of a normal pregnancy when serum hCG concentration peaks (9–11 weeks of pregnancy). GH is generally mild, asymptomatic, and self-limiting. Hyperemesis gravidarum occurs in 1.5% of pregnancies and is characterized by hyperemesis, dehydration, electrolyte abnormalities, and hyperthyroidism (~ 30–40%) [11–13].

hCG is a glycoprotein hormone with structural similarity to TSH that is composed of a common alpha subunit [shared by all glycoprotein hormones (FSH, LH, TSH)] and a hormone-specific beta subunit. Interestingly, the beta subunits of both hCG and TSH are also remarkably homologous: both contain strategically located cysteine residues and disulfide bonds that form a cysteine knot structure, crucial for receptor binding [12, 14, 15].

hCG is capable of inducing hyperthyroidism when hCG levels are extreme and sustained for several weeks. Hyperthyroidism can occur with hCG levels > 200 mIU/mL, but overt hyperthyroidism is usually seen in germinal tumors when hCG concentration is >50,000−100,000 mIU/mL [12, 15–19]. Roughly, 25,000 U/L of hCG is equivalent to 1 mU/L of TSH activity [14, 15]. The level of thyroid stimulation is directly proportional to hCG concentration, but not all patients with elevated hCG levels present with hyperthyroidism; this is thought to depend on secondary modifications of the hCG molecule, such as glycosylation and sialylation, that affect hCG bioactivity with differing TSH receptor sensitivity. hCG deglycosylation and desialylation may promote thyrotropic potency [12, 16, 20, 21]. Moreover, hCG produced by GTD is usually more potent than normal pregnancy hCG because of the lower sialic content of GTD hCG; < 3% of the oligosaccharide side chains of hCG derived from a choriocarcinoma contain sialic acid, whereas normally pregnancy-produced hCG is mostly sialylated [12, 21].

In some cases of extreme paraneoplastic hCG production, a “hook effect” leads to falsely low and misleading measured serum concentration. In a uterine choriocarcinoma case report, initial measurement of hCG concentration was 193 mU/mL with a real value after serial dilutions of > 11 million mIU/mL [16]. In our patient, serial hCG dilutions were accordingly performed, confirming very high hCG concentration (~ 2 million).

The 2016 American Thyroid Association (ATA) guidelines [11] acknowledge trophoblastic disease/choriocarcinoma as a cause of thyrotoxicosis, characterized by diffuse hyperactive thyroid. The guidelines, furthermore, recognize that thyrotoxicosis can occur in the setting of choriocarcinoma due to “the effect of tumor-derived hCG upon the TSH receptor. This cross-stimulation only occurs at very high levels of hCG, since hCG is only a weak agonist.”

However, it is perhaps underappreciated that hCG-induced hyperthyroidism may be life threatening if not recognized promptly. Treatment usually consists of correcting the cause, if possible. In the choriocarcinoma setting, effective cancer management decreases hCG concentration, palliating thyrotoxicosis. Nonetheless, overt hCG-induced hyperthyroidism requires medical therapy with antithyroid drugs and beta-blockers, with some patients requiring Lugol’s solution in parallel. The 2016 ATA guidelines suggest that treatment of hyperthyroidism due to choriocarcinoma should include both methimazole as well as treatment directed against the primary tumor [11]. There are, however, no specific recommendations on hyperthyroidism management in the setting of an extragonadal widely metastatic choriocarcinoma with imminent liver failure.

Our patient presented with very symptomatic hyperthyroidism along with multiple liver masses, requiring treatment with methimazole and propranolol and briefly Lugol’s solution, with adequate response to therapy and resolution of thyrotoxicosis.

A remarkable feature of our patient’s neoplastic cells was the strong and diffuse membranous reactivity for the programmed cell death ligand-1 (PD-L1) antibody (Fig. 2). This, although striking, is consistent with previous reports. In an immunohistochemical study of PD-L1, it was demonstrated that it was constitutively and strongly expressed in normal tissue placental trophoblast, choriocarcinomas, and trophoblastic components of germ cell tumors. Choriocarcinomas were uniformly positive for PD-L1 with a membrane pattern, suggesting that PD-L1 might be a supplemental marker for diagnosis of choriocarcinoma or trophoblastic features in other germ cell tumors [22]. A study assessing in detail the distribution of PD-L1 expression in human placentas and GTD showed that PD-L1 was highly expressed in syncytiotrophoblast in complete moles and choriocarcinomas [23].

High PGC PD-L1 expression suggests that cancer treatment with immune checkpoint inhibitors may be fruitful, with a case report showing a partial response to nivolumab as second-line treatment for poorly differentiated adenocarcinoma with choriocarcinomatous features [24].

Choriocarcinoma is usually chemosensitive and has excellent outcomes when gonadal in origin [2, 3]. However, PGC has dismal prognosis; an analysis of 53 patients showed that 19% died < 1 month postoperatively [4] with median OS of < 2 months [5–7]. There is no standard of care for PGC patients, but commonly used chemotherapeutic agents include, methotrexate, etoposide, folinic acid, actinomycin D, cyclophosphamide, vincristine, and others [2, 25]. Our patient received initially methotrexate with no response and later etoposide-cisplatin with biochemical decrease of hCG tumor marker but with structural progression and ultimate liver failure–multiple organ failure. One wonders whether PGC location might be the main driver of poor prognosis, since a gastric location of a highly vascularized “placental-like” tumor with rapid growth and hematogenous spread entails easy access to hepatic seeding with consequent widespread liver metastatic disease upon diagnosis, many times with little normal liver reserve, with all the calamities associated with this context. Moreover, thyrotoxicosis treatment and need for chemotherapy in the setting of high tumor burden and hepatic failure is particularly challenging.

Univariate and multivariate analysis of PGC pooled data showed that the presence of residual tumor, synchronous liver metastasis, and absence of chemotherapy were significantly associated with increased mortality [4]. Our case had widespread synchronous liver metastases with high tumor burden. The most frequent causes of death in PGC are hepatic failure due to metastatic spread (29%) and bleeding from primary or metastatic disease (24%). Our patient died of both complications.

## Conclusion

Primary gastric choriocarcinoma (PGC) is an aggressive malignancy with very poor outcomes. The co-occurrence of severe thyrotoxicosis with advanced PGC and imminent liver failure complicates management options. We believe that this case is of educational value and that it might aid future PGC patients and clinicians, favoring early recognition and expedited care, as well as to set expectations clear upfront of the prognosis of this terrible disease complicated by severe thyrotoxicosis. Finally, we hope that this report will lead to the design of research studies for future therapies that could improve the grim prognosis of this malignant disease.

## Data Availability

Data sharing is not applicable to this article as no datasets were generated or analyzed in this single-patient case report.
